# Comparison of two instruments for measurement of quality of life in clinical practice - a qualitative study

**DOI:** 10.1186/1471-2288-14-115

**Published:** 2014-10-09

**Authors:** Lena Wettergren, Mathilde Hedlund Lindberg, Åsa Kettis, Bengt Glimelius, Lena Ring

**Affiliations:** Department of Neurobiology, Care Sciences and Society, Division of Nursing, Karolinska Institutet, Huddinge, Sweden; Centre for Research Ethics & Bioethics, Uppsala University, Uppsala, Sweden; Department of, Radiology, Oncology and Radiation Science, Uppsala University, Uppsala, Sweden; Department of Oncology and Pathology, Karolinska Institutet, Stockholm, Sweden

**Keywords:** Cognitive interviews, Gastrointestinal cancer, Health-related quality of life, Measures, SEIQoL, Quality of life

## Abstract

**Background:**

The study aimed to investigate the meaning patients assign to two measures of quality of life: the Schedule for Evaluation of Individual Quality of Life Direct Weighting (SEIQoL-DW) and the SEIQoL-DW Disease Related (DR) version, in a clinical oncology setting. Even though the use of quality of life assessments has increased during the past decades, uncertainty regarding how to choose the most suitable measure remains. SEIQoL-DW versions assesses the individual’s perception of his or her present quality of life by allowing the individual to nominate the domains to be evaluated followed by a weighting procedure resulting in qualitative (domains) as well as quantitative outcomes (index score).

**Methods:**

The study applied a cross-sectional design with a qualitative approach and collected data from a purposeful sample of 40 patients with gastrointestinal cancer. Patients were asked to complete two measures, SEIQoL-DW and the SEIQoL-DR, to assess quality of life. This included nomination of the areas in life considered most important and rating of these areas; after completion patients participated in cognitive interviews around their selections of areas. Interviews were audiotaped and transcribed verbatim which was followed by analysis using a phenomenographic approach.

**Results:**

The analyses of nominated areas of the two measures resulted in 11 domains reflecting what patients perceived had greatest impact on their quality of life. Analysis of the cognitive interviews resulted in 16 thematic categories explaining the nominated domains. How patients reflected around their quality of life appeared to differ by version (DW vs. DR). The DW version more often related to positive aspects in life while the DR version more often related to negative changes in life due to having cancer.

**Conclusions:**

The two SEIQoL versions tap into different concepts; health-related quality of life, addressing losses and problems related to having cancer and, quality of life, more associated with aspects perceived as positive in life. The SEIQoL-DR and the SEIQoL-DW are recommended in clinical practice to take both negative and positive aspects into account and acting on the problems of greatest importance to the patient.

## Background

Measurement of quality of life (QoL) in clinical practice is receiving increasing interest. It is, however, essential that the understanding and interpretation of these assessments are based on patients’ perceptions and that the knowledge is shared with health care personnel [1, 2] as well as policy makers and patients. Patient-reported outcome measurements (PROMs), e.g. QoL measures, are commonly used in medical research including randomized clinical trials [3]. However, they are less common when monitoring patients in clinical practice [4–6] nevertheless recognized as important [7]. Even though the use of QoL assessments has increased during the past decades, problems regarding how to choose the most suitable measurement remains [7]. There are several existing PROMs assessing health-related quality of life (HRQOL) or disease symptoms in various patient populations. Many of these measures are based on a generalized, nomothetic approach opting for group comparisons [8]. These type of measures have been criticised for their pre-set domains, enhancing the risk of missing domains essential for the individual’s QoL or including domains of less importance [8]. In contrast to this approach, individualized measures have been developed to capture the concept of QoL from an idiographic view, i.e., taking into account the uniqueness of the human experience [9, 10].

A few individualized measures exist, e.g., the Patient Generated Index (PGI) [11] and the Schedule for Evaluation of Individual Quality of Life - Direct Weighting (SEIQoL-DW) [12] developed from the original SEIQoL [13]. The SEIQoL-DW has proved to be a valid and reliable measure to capture the perceived QoL in various patient populations including those severely ill [10, 14]. All SEIQoL versions assesses the individual’s perception of his or her present QoL and allows the individual to choose the domains to be evaluated followed by a weighting procedure resulting in qualitative (domains) as well as quantitative outcomes (index score) [12]. There is also a more recently developed disease-related version, SEIQoL-DR [15], directing attentiveness towards the individual’s perception of her/his well-being *in relation to having an illness* when nominating the domains most important for QoL [14, 16]. Results from the two versions have found differences in index scores with higher scores, reflecting better QoL, for SEIQoL-DW compared to the SEIQoL-DR [14, 17]. However, the nominated domains are about the same, although those nominated using the SEIQoL-DR tend to be lower rated [17] and a possible explanation could be that despite considering, for instance, family relations as crucial in life and therefore rated high with the SEIQoL-DW, the domain may also be related to strain on family relations due to disease and treatment, reflected in a lower rating when using the SEIQoL-DR. This indicates that the two instruments may tap into different notions of QoL [17]. However, how patients reason when nominating cues is not known and information about the reasoning that leads to the patients’ answers will contribute to a deeper understanding of possible needs of health care. If the instruments are clearly differentiated in what they capture, and their respective qualities are ruled out, it is possible to make a choice of which version to use in a specific context, and how better to interpret the results. The aim of this study was, therefore, to explore how gastrointestinal (GI) cancer patients reason when nominating domains using the SEIQoL-DW and the SEIQoL-DR, i.e. when being directed to focus on overall QoL compared to being asked to focus on how life is affected by having cancer.

## Methods

The study employed a cross-sectional design with a qualitative approach based on cognitive interviews and standardised measures.

### Measures

#### SEIQoL-DW

The participants were first asked to nominate their five most important QoL domains (cues) and also rate their current status in each domain on a visual analogue scale ranging from 0 (worst possible) to 100 (best possible) [12]. In the last stage, the relative importance of the areas were taken into account by a weighting procedure. The patients were asked to quantify the importance of each area, represented by five differently coloured areas of a pie chart, by adjusting the sizes of the identified life areas. All areas add up to 100, and the area perceived to be of greatest importance should subsequently be assigned the largest pie area. Both versions produce an overall QoL index score to enable comparisons at the group level; the index score is calculated by multiplying the rating of each area with the same domain’s weight, and then adding the products according to standard procedures (range 0–100), with a higher score reflecting a better outcome [12]. Computer-administrated versions of the SEIQoL-DW and SEIQoL-DR were used which have been shown to have acceptable feasibility and validity [9, 17].

#### SEIQoL-DR

The participants were asked to nominate their five most important QoL domains influenced positively and negatively by having GI cancer. The procedures for nominating the current status and the weighting of the domains were the same as described above regarding SEIQoL-DW.

### Participants

In order to capture a variety of perspectives, i.e. achieve heterogeneity, a purposefully selected sample of 40 patients (20 men and 20 women) were invited to take part in the study based on the following criteria: Swedish-speaking men and women of different ages willing to take part in the study, from two healthcare regions in Sweden, diagnosed with GI cancer and treated at two different university hospitals. Data were collected during five months during 2004. Out of 40 patients, 29 had colorectal cancer, 4 anal cancers, 3 pancreatic cancers, 2 hepato-biliary cancers and 2 gastric cancers. Thirty-five were on chemotherapy or radio- (chemo) therapy, while five had supportive care only. The purposes of the treatments were curative in 11 patients (colorectal cancer, n =8; anal cancer n =3) and palliative in 29 patients (colorectal cancer, n =24). The median survival is about 20 months for colorectal cancer patients if actively treated with chemotherapy. For the remaining diagnoses and for patients not actively treated, median survival is much shorter, approximately 3–8 months. The median age was 59 (33–78) years.

### Procedures and analysis

Potential participants were approached by a research nurse in the clinic, when receiving regular treatment or at follow-up, and orally informed about the study. Additionally, patients also received a letter with information about aims and procedures of the study which stressed that participation was voluntary and confidential. Oral informed consent was obtained from all participants prior to inclusion in the study. The study was approved by the Regional Ethics Committee for Human Research at Uppsala University (No 2004:M-025). The presented study is reported in accordance with the STROBE guidelines for cross-sectional studies.

The participants completed both versions of the SEIQoL on a touchscreen computer immediately before a medical consultation. All patients first filled in the SEIQoL-DW followed by the DR-version. The participating patients nominated a total of 200 cues per instrument. These cues were aggregated, based on similar meanings, by the researchers into 11 different domains (see Table 1).

The first 20 participants were exposed to a concurrent interview, i.e. audiotaped while completing the questionnaires and encouraged to “think aloud” [18]. The “think aloud” method is commonly used in order to encourage research participants to express their thoughts out loud while answering questionnaires. The benefit of a concurrent “think aloud” interview is that it provides insight into the respondents’ thinking process [18]. Due to some criticism regarding artificiality associated with concurrent “think aloud” interviewing [18], a combined approach was used. The remaining half of the participants (n =20) where audiotaped while completing a retrospective cognitive interview with probing immediately after completing the SEIQoL versions. There was no random allocation to either group, only equal distribution due to sex.

**Table 1:** **Domains based on patient’s nomination of important life areas and corresponding thematic categories**

Domains	SEIQoL-DW N = 200 ^1^ (100%)	SEIQoL-DR N = 200 ^1^ (100%)	Thematic categories	Original SEIQoL version
Relationships	56 (28)	43 (21.5)	Respect and cherish	DW
			Social relationships changed by cancer	DR
Health	39 (19.5)	46 (23)	Cancer related to body and mind	DW, DR
Leisure	37 (18.5)	29 (14.5)	Leisure	DW
			Leisure limited by cancer	DR
Finances	24 (12)	33 (16.5)	Financial difficulties	DW, DR
Social activities	23 (11.5)	26 (13)	Supportive network	DW
			Inability to meet with friends	DR
Physical activity	11 (5.5)	13 (6.5)	Strength from exercise	DW
			Losing ability to exercise	DR
Independence	4 (2)	4 (2)	Leading an independent life	DW
			Limited independence	DR
Living conditions	3 (1.5)	1 (0.5)	Peaceful environment	DW, DR
Mental strength	1 (0.5)	4 (2)	Mentally stronger	DW, DR
Religiosity	1 (0.5)	1 (0.5)	Comfort in religious beliefs	DW, DR
Writing memoirs	1 (0.5)	0	Passing on family history to next generations	DW

^1^Each patient nominated five cues summing up to 200 cues/instrument; all domains consist of aggregated cues.

All interviews were listened to several times and transcribed verbatim. One concurrent interview was excluded because it mainly contained discussions on the difficulty of dealing with the computer. A phenomenographic approach, which emerged from an empirical base [19], was applied to clarify the qualitative variations in how patients reason when nominating domains depending on which of the two instruments were used. Given the heterogeneity of the participants in this study and the combination of concurrent and retrospective “think aloud” approaches, a relatively high number of patients were included [20]. This means that a wide range of ways of reasoning about the cues among GI cancer patients was likely to have been covered.

The audiotaped interview sessions (concurrent and retrospective) were analysed separately for the SEIQoL-DW and SEIQoL-DR, but the findings are presented together. All text analyses were carried out using the software program QSR NUD*IST VIVO^®^ (Version 1.2).

## Results

The median amount of time spent on filling in the SEIQoL-DW and SEIQoL-DR was five minutes (2–12) and seven minutes (2–17), respectively. Not surprisingly, the concurrent “think aloud” interviews contained more discussions about technical computer-related matters than the retrospective interviews. Accordingly, the participants had a slightly more reflective approach towards the research topic during the retrospective interviews than during the concurrent “think aloud” interviews.

**Figure 1:**
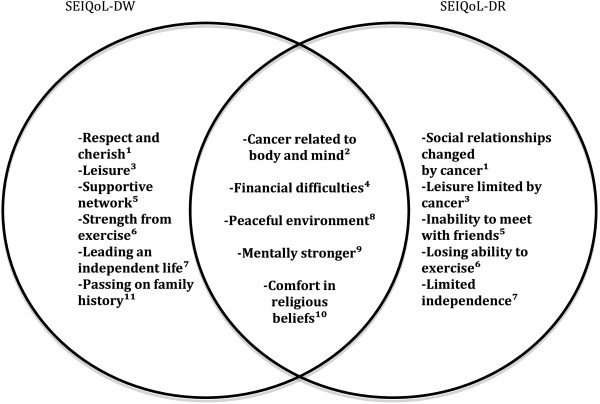
**Thematic categories reflecting thoughts about nomination of domains crucial for quality of life.** Exclusive for SEIQoL-DW (left), common categories (middle), exclusive for SEIQoL-DR (right). Related domain names: Relationship^1^, Health^2^, Leisure^3^, Finances^4^, Social activities^5^, Physical activity^6^, Independence^7^, Living conditions^8^, Mental strength^9^, Religiosity^10^, and Writing memoirs^11^.

The SEIQoL nomination procedure resulted in 11 domains based on the respondents’ completion of the instruments, i.e. cue nomination (Table 1). Further, the analysis of the “think aloud” and retrospective cognitive interviews regarding nomination of domains resulted in 16 thematic categories, which are listed in Table 1 in relation to the domain which they further explain. In Figure 1, the thematic categories are shown based on the originating instrument. Each domain and thematic category will be presented below and illustrated with selected quotes.

### Relationships

The participants’ thoughts and reasoning when nominating *relationships* resulted in two thematic categories reflecting the difference in reasoning, i.e. ‘respect and cherish’ (SEIQoL-DW) and ‘social relationships changed by cancer’ (SEIQoL-DW). Some participants answering the SEIQoL-DW expressed an altered view and increased respect for other people that they had developed, in contrast to before falling ill. Furthermore, the closest family members were thought of as important for emotional support and should, therefore, be cherished. The participants also included pets as family members, cherishing the perceived silent companionship with the animals.

*P12: . . . I think about the family you live with, together with your partner and children. It is much more important than many people understand and think it is. I mean it is together with them you do everything. It is in the family where you get support if you need support. It is with the family that you can cry and laugh. It is both from your children and your life companion that you can gain so much incredible support and help when you need it. So, the family you have to be really careful with.*

Female (SEIQoL-DW, think aloud)

The participants’ thoughts when answering the SEIQoL-DR were related to changes in relationships. Patients described how the closest family and friends were affected by their cancer and, in some cases, relationships had ended due to the situation. Still, the majority described an overall positive impact and felt closer to their family, even though the family burden increased as a result of the disease. Some individuals acknowledged fear of permanently losing work mates and good friends. A positive relationship with the hospital staff was emphasised by some participants.

*P15: Yes, and it is the family, too. It has an impact on them also. When I get treatment they have to help along (/…./). Of course it has an impact on them and they cannot really do anything*.

Female (SEIQoL-DR, think aloud)

### Health

The participants’ thoughts about nominating *health* resulted in one category, i.e. ‘cancer related to body and mind’*.* The statements regarding health reflected a change in the perspective of physical health. Before being diagnosed with cancer, it was not thought about at all; however, after diagnosis, they were constantly reminded about their health. Thoughts about well-being, also included in the health domain, were associated with positive features such as being alive, peace, inspiration and beautiful surroundings. While physical health was mostly associated with negative features such as pain, sleep disturbances and loss of autonomy. Some of the participants linked these negative features to the side effects of treatment (fatigue, changed sexuality). Also, the inability to plan for tomorrow was expressed, together with the inability to trust one’s own health and body, and never knowing if the cancer had spread as illustrated by one patient:

*P52: To me, health is that you have strength to get out of bed in the morning and do things during the day. And I can’t . . . you can never presume and plan anything like tomorrow we do this or that. . .*

Male (SEIQoL-DW, think aloud)

There were similarities between thoughts when nominating with the SEIQoL-DW and the SEIQoL-DR, especially regarding thoughts for nominating well-being. There were also some differences; for example, the inability to plan ahead was only mentioned when using the SEIQoL-DW.

*P50: Yes, well, it’s all about you feeling well and being fine; when I do, then I am happy. But how I feel affects me very much, and when I’m not feeling so well and I’m tired, then I don’t have the energy to do things I usually do and I feel pretty bad (/…./)*

Female (SEIQoL-DR, think aloud)

### Leisure

The participants’ thoughts about nominating *leisure* resulted in two thematic categories, i.e. ‘leisure’ and ‘leisure limited by cancer’*.* The nominated areas with the SEIQoL-DW that were considered important highlighted the ability to relax from work. Commonly, thoughts around activities carried out in their free time were expressed, such as choir singing, gardening, boating and wandering in the outdoors. Having GI cancer was not mentioned.

When nominating important domains using the SEIQoL-DR, free time was associated with limitations due to the cancer; the inability to travel due to treatment was stressed by several participants. The limited possibility to spend holidays and free time as desired was described as intruding on life:

*P52: We play golf and that is impossible to do when you have ongoing chemotherapy. You cannot go abroad to play.*

Male (SEIQoL-DR, think aloud)

### Finances

The participants’ thoughts about nominating *finances* resulted in one category, i.e. ‘financial difficulties’. This category had become more and more important during the course of the disease since long sick leave had resulted in a difficult financial situation and, in some cases, quite a strain. There were no variations in the statements by the SEIQoL-DW version used.

### Social activities

The participants’ thoughts about nominating *social activities* resulted in two thematic categories, i.e. ‘supportive network’ and ‘inability to meet with friends’. When filling in the SEIQoL-DW, the participants thought about social networks, stressing the importance of friends and colleagues for support and companionship. Taking part in recurrent social activities was considered important. While the inability to maintain contact with, and even lose friends due to cancer, was featured in this category when answering the SEIQoL-DR (“inability to meet with friends”). Also, the inability to participate in social activities was acknowledged.

*P50:I’m too tired to meet them (friends) when I get chemotherapy; I don’t even have the energy to speak over the phone .... all that easy going and fun are gone…*

Female (SEIQoL-DR, think aloud)

Some participants stressed that they thought they were treated differently by friends because of their cancer, when engaging in social activities.

### Physical activities

The participants’ thoughts about nominating *physical activities* resulted in two thematic categories, i.e. ‘strength from exercise’ and ‘losing ability to exercise’, reflecting variations in how the participants thought when nominating physical activity, i.e. having the strength to fight the cancer (DW) and losing the ability to perform exercise (DR). Answers to the SEIQoL-DW were associated with athletics and other forms of exercise to try to gain strength to fight the cancer. The participants stressed that they actually felt better from exercising. In reflecting aloud when answering the SEIQoL-DR (‘losing ability to exercise’), the participants only mentioned exercise in relation to the loss and inability to perform activities (sports, gymnastics) they had done before the cancer:

*P14:To exercise has not worked, of course. The week when I get treatment, if you can’t breathe, you can’t be outdoors. It is impossible.*

Female (SEIQoL-DR, think aloud)

### Independence

The participants’ thoughts about nominating *independence* resulted in two thematic categories, i.e. ‘leading an independent life’ and ‘limited independence’. The thoughts while filling in the SEIQoL-DW were more directed towards the individual’s life outside the hospital, whereas the thoughts when filling in the SEIQoL-DR were related to having cancer. The participants cherished their independence and freedom when answering the SEIQoL-DW. The thoughts on their independence were, however, not specifically associated with having cancer, but with life in general and with work.

*P12:* . . . *independence is . . . to live and to be able to do everything you want to and not being dependent on another person.*

Female (SEIQoL-DR, think aloud)

When answering the SEIQoL-DR, independence was associated with limitations due to negative changes related to the GI cancer. Furthermore, thoughts about being a burden on family members were acknowledged. A female patient expressed her dependence on her husband:

*P14:* . . . *with this disease . . . you are not independent. You have to . . . my husband has to do everything*.

Female (SEIQoL-DR, think aloud)

### Living conditions

The participants’ thoughts about nominating *living conditions* resulted in one category, ‘peaceful environment’. Living in a peaceful, quiet environment was considered important for well-being. There was no variation among the answers categorized in this domain.

### Mental strength

The participants’ thoughts about nominating *mental strength* resulted in one category, i.e. ‘mentally stronger’. Some of the participants meant that having to deal with the cancer had made them mentally stronger compared to before being diagnosed with cancer. They also considered themselves calmer and more peaceful due to the increased mental strength. There were no variations between the SEIQoL versions used by the participants.

*P11: I perceive myself as much calmer since I got this; before I could be afraid of death and that’s all gone . . . I am calm and in harmony.*

Male (SEIQoL-DW-DR, retrospective)

*P51: Yes . . . it’s just that you feel okay. You give yourself this time to get to know yourself honestly.*

Female (SEIQoL-DW, think aloud)

### Religiosity

The participant’s thoughts about nominating *religiosity* resulted in one thematic category, i.e. ‘comfort in religious beliefs’. A personal religious belief was thought of as a source for giving comfort and hope. The respondents did not, however, report that their religious beliefs were decreased or negatively altered by experiencing the burden of having cancer and undergoing treatment.

### Writing memoirs

The participant’s thoughts about nominating *writing memoirs* resulted in one thematic category. The writing memoirs domain was only nominated based on the SEIQoL-DW, and was considered important in order for children and grandchildren to have information about their family history, and as a way of letting them know their parent/grandparent more intimately. The approaching death was also a key element when nominating this domain.

*P28:* . . . *passing on family history to the next generations.*

Female (SEIQoL-DW, retrospective)

## Discussion

Analysis of cognitive interviews of the participants’ reasoning when answering the generic SEIQoL-DW and the disease-related SEIQoL-DR resulted in 16 thematic categories explaining the nominated domains. The patients’ reflections around their health appear to differ between the two versions. The generic version (DW) was more often related to positive aspects in life, while the DR version was more often related to negative changes in life due to cancer. Further studies are needed to evaluate if SEIQoL cues, from both versions, might be useful in relation to existing frameworks and theories, for example, coping strategies, response shift, resilience and life span health.

The *relationship* domain was associated with cherishing relationships (DW-version), in contrast to the loss of significant others due to having cancer (DR-version), pointing out that the two SEIQoL versions tap into diverse aspects in this domain. This is in line with a previous quantitative study indicating that the same QoL domains were rated differently in the SEIQoL-DW and the SEIQoL-DR [17]. The ability to preserve significant interpersonal relationships appears to be important, and previous research has associated the individual’s capacity to maintain such relationships with good functioning and well-being [21]. Having their ‘social relationships narrowed by cancer’ could, in that respect, put patients at risk for lower QoL/HRQOL. Consequently, ‘respect and cherish’ could be considered a health resource as opposed to the loss of meaningful relationships. The nomination of the *leisure* domain was also associated with different features for the two versions. When using the SEIQoL-DW, the participants did not make any associations with their cancer, in contrast to filling out the SEIQoL-DR. In the DW version, *leisure* was associated with being outdoors. Previous research has found that interactions with nature, such as gardening, are essential for well-being [22]. *Physical activity* is another domain of interest, since studies have shown the effect of physical exercise in combination with behavioural support to increase the QoL in patients with cancer [23]. Some thematic categories overlapped between the two instruments, e.g. ‘financial difficulties’ and being ‘mentally stronger’. The findings presented under ‘financial difficulties’ indicate a negative impact on patients with GI cancer, i.e. their disease and treatment affected their economy which, in turn, affected their perceived well-being. This was seen for both instruments used.

The nomination of domains was not always associated with negative features due to having GI cancer; for instance, the category ‘mentally stronger’ (both instruments) constituted the ability to get to know oneself, feeling calmer and being mentally strong. This is in line with a previous study of survivors of ovarian cancer linking protecting factors of resilience and growth to QoL [24]. The awareness of capacities for personal development to deal with physical and mental health decline is important for the provision of treatment and care of high quality [25]. This has also been put forward in work related to a psychological well-being approach, where domains such as self-development, personal growth and purposeful engagement are included [26]. One approach is the selection, optimization, and compensation (SOC) model, a complex meta-model outlining strategies for how a person can select and pursue goals in accordance with changing personal, biological and social contexts [27]. A questionnaire with subscales assessing selection (goal setting), optimization (internal and external resources) and compensation (maintaining goals) has been developed for use throughout the lifespan to investigate strategies people use to adapt to life circumstances. The potential to use SEIQoL for identifying specific domains which might further be acted on or to base individual interventions on SOC strategies need to be studied in future research.

The findings of the study open up a discussion regarding the utility of QoL versus HRQOL as concepts and the usefulness of assessing these in clinical practice. The results indicate that the SEIQOL-DR version taps into HRQOL while the SEIQOL-DW version taps into QoL which is in line with results concerning standardized measures [28]. Patients’ perceptions of nominating some of the QoL domains using the SEIQoL-DW were shown to be associated with protective factors, i.e. aspects of an individual’s life associated to well-being, which healthcare providers do not usually attend to. Some of the domains brought up when using the SEIQoL-DR might, on the other hand, were associated with disease-related symptoms or problems that usually encumber well-being and health. In this perspective, using SEIQoL-DW in combination with disease-related instruments, such as the SEIQoL-DR, to focus on problems and symptoms in need of healthcare actions may provide nurses and other healthcare professionals with information on individual patient’s health resources, and suggest ways of empowering patients, in this case GI cancer patients, to increase their overall well-being. The procedure and results of both SEIQoL versions are intuitively easy to understand, for patients as well as for clinicians, making them feasible for use in clinical practice.

There are some limitations in the present study. By not varying the order of filling in the instruments, we do not know if that may have had an influence on the results. Furthermore, one interview had to be excluded due to the participant experiencing difficulties using the computer. The present study could be criticized in the same way as previous studies using the “think aloud” method, by creating a false milieu with a researcher present in the room and, in addition, that the participants have to talk aloud, which they normally do not do [18]. However, in order to meet this criticism, we also used a retrospective interview. The benefit of cognitive interviewing is that it accurately captures how patients understand the questions in a questionnaire; thus, in the present study, their view of QoL/HRQOL, i.e. content validity. In order to strengthen the trustworthiness regarding *credibility*, the researchers chose a heterogeneous sample, which is suggested to contribute to a richer variation of perspectives [29]. Eleven of the patients were treated in connection with the primary diagnosis with a statistical chance of cure ranging from low to very high depending upon tumour stage. Most patients were in a palliative phase, however, with very variable life expectancy from a few months to some years. None of the patients included were in a terminal phase with an expected survival of only one or two months. Furthermore, two of the co-researchers used a consensus procedure to reach agreement about the categorisation. It cannot be ensured that saturation was reached regarding some of the domains since they were nominated on one occasion by the same individual in both versions, i.e. religiosity. However, the findings regarding the positive aspects dominating in relation to QoL and more problem-oriented aspects directed towards HRQOL, has been suggested in previous research of patients undergoing stem cell transplantation [30]. To increase *dependability*, the interviewer used a semi-structured interview guide when performing the retrospective interviews [29].

## Conclusions

There is a qualitative variation in individuals’ thoughts and reasons for nominating several of the most important domains, when filling in the SEIQoL-DW compared to the SEIQoL-DR. The findings support the assumption that the two instruments tap into different concepts. The thematic categories related to SEIQoL-DR more often addressed problems that may be in need of healthcare actions than those related to the SEIQoL-DW. Furthermore, it is hypothesised that the thematic categories related to SEIQoL-DW are associated with protective factors, i.e. health resources, and the SEIQoL-DR with health hindrances. Resources may be aspects of an individual’s life that are related to well-being and/or joy, but not normally addressed by standard healthcare. However, identifying unique health resources that give meaning to the individual during illness could be useful in patient counselling to cope with the burden of treatment. This may be of relevance when implementing SEIQoL-DR and/or SEIQoL-DW in clinical practice.

The SEIQoL-DR and the SEIQoL-DW are recommended for use in clinical practice to take advantage of the patient’s resources and act on the problems of greatest importance to the patient. In this way, care could focus on strengthening the patient’s resources and acting on perceived obstacles.

## Abbreviations

GI: Gastro Intestinal
HRQOL: Health-related quality of life
PGI: Patient generated index
PROMs: Patient reported outcome measures
QOL: Quality of life
SEIQoL-DW: The schedule for individual quality of life – direct weighting
SEIQoL-DR: The schedule for individual quality of life – disease related.
